# Loneliness and personal well‐being in young people: Moderating effects of individual, interpersonal, and community factors

**DOI:** 10.1002/jad.12046

**Published:** 2022-04-10

**Authors:** Claire Goodfellow, Deborah Hardoon, Joanna Inchley, Alastair H. Leyland, Pamela Qualter, Sharon A. Simpson, Emily Long

**Affiliations:** ^1^ MRC/CSO Social and Public Health Sciences Unit University of Glasgow Glasgow UK; ^2^ What Works Centre for Wellbeing London UK; ^3^ Manchester Institute of Education University of Manchester Manchester UK

**Keywords:** loneliness, moderation, social ecological, wellbeing, young people

## Abstract

**Introduction:**

Loneliness is prevalent among young people. But, there is little work exploring the association between loneliness with well‐being among this age group. Framed by social‐ecological theory, we examined demographic, interpersonal, and community factors associated with personal wellbeing and, critically, identified malleable moderators of the relationship between loneliness and well‐being that could be targeted in intervention efforts.

**Methods:**

We used cross‐sectional, secondary data from 965 young people (aged 16–24) from the Community Life Survey in England. Loneliness was measured using a single‐item direct measure; personal wellbeing was measured through a composite measure containing items assessing happiness, life satisfaction, and a sense that life is worthwhile (*α* = 0.88). Regression techniques were used to assess associations between individual, interpersonal, and community factors and well‐being, and to identify moderators of the relationship between loneliness and well‐being.

**Results:**

Loneliness was negatively associated with well‐being. Chatting with neighbors and having people to provide help moderated the relationship between loneliness and well‐being. Full‐time students and those with good physical health had higher well‐being while being a carer was predictive of lower well‐being. All community variables were strongly associated with increased well‐being. Of all interpersonal variables investigated, only having people to count on was associated with increased well‐being.

**Conclusions:**

Our results demonstrate that supportive relationships and close community ties are important for reducing the negative impact of loneliness on youth well‐being. Interventions to improve well‐being could benefit from targeting these aspects of young people's social and community lives, while acknowledging individual vulnerabilities, such as poor physical health.

## 1 INTRODUCTION

Loneliness is an international public health concern and represents a significant public health challenge (Department for Digital Culture Media and Sport, 2018; Gerst‐Emerson & Jayawardhana, 2015). Loneliness affects people of all ages, although it is especially prevalent among young people aged 16–24 years (Office for National Statistics, 2018). The consequences of loneliness on physical health, well‐being, and mortality are recognized as priority areas for public health research and policy development (Department for Digital Culture Media and Sport, 2018; What Works Centre for Wellbeing, 2019), with their relevance particularly acknowledged since the Covid‐19 pandemic.

Loneliness is typically defined as the negative, emotional experience that occurs due to a perceived absence of desired social relationships (Cacioppo & Hawkley, 2009; Perlman & Peplau, 1981), either in terms of quantity or quality (Qualter et al., 2015). It is important to note that the subjective experience of loneliness is not synonymous with the objective experience of being alone, or the quantifiable absence of close relationships, otherwise known as objective isolation (de Jong Gierveld & Havens, 2004). For example, it is possible to experience subjective loneliness when surrounded by other people, or when alone.

Loneliness is common among young people (Office for National Statistics, 2018; Qualter et al., 2015), but most research examining loneliness looks at the experience among older adults or school‐aged adolescents (Qualter et al., 2015). That means there is a lack of research that examines loneliness among older adolescents (16–24 years of age) specifically (Luhmann & Hawkley, 2016; Victor & Yang, 2012). Where robust population‐based evidence does exist, it demonstrates that loneliness among older adolescents is associated with poorer mental health, including an increased risk of depression, anxiety, ADHD and conduct disorder, negative coping strategies, such as self‐harm, social skills deficits, and increased aggression (Ladd & Ettekal, 2013; Matthews et al., 2019; Schinka et al., 2013). However, that research was limited to an examination of loneliness among those aged only 18 years. Other work with school‐aged adolescents shows similar relationships (Yang et al., 2022), but the experience of loneliness during the compulsory school years may be particularly contextualized (Jefferson et al., 2022), and may not be transferable to understanding the experience of loneliness among older adolescents. As such, although adolescents 16–24 years of age are particularly prone to experiencing loneliness, less is known empirically about the effects of loneliness in this age group.

### 1.1 Loneliness and personal wellbeing

There are many conceptualizations of well‐being. In this study, we focus on personal well‐being (PWB) defined as individuals', “good mental states, including all of the various evaluations, positive and negative that people make of their lives and the affective reactions of people to their experiences” (Diener, 2006; OECD, 2013). PWB has the potential to provide a protective effect against poor health and has been shown to reduce the use of health services (Keyes, 2007) and improve longevity (Diener & Chan, 2011; Diener et al., 2017). Due to the noted benefits of PWB, many countries now promote positive wellbeing among young people as a national policy priority (e.g., Clarke et al., 2011; Mental Health and Wellbeing EU Joint Action, 2017; Väilmaa et al., 2007).

Consistent evidence links loneliness and personal wellbeing among older adults (Chen & Feeley, 2014; Holt‐Lunstad et al., 2015; Tomaz et al., 2021), with research often exploring how loneliness mediates the association between poor social relationships (quality, number) and well‐being (Arslan, 2021; Lasgaard et al., 2011). While such work is important, generalizing findings from research with older adults to young people is problematic, given that experiences of loneliness are known to vary in different demographic groups (Franssen et al., 2020) and young people inhabit different developmental and social contexts.

Comparatively little is known about the relationship between loneliness and PWB among older adolescents, and the work with school‐aged adolescents rarely uses large representative datasets, making the generalization of findings difficult. Thus, given the acknowledged importance of PWB to ongoing public health initiatives (Clarke et al., 2011; Mental Health and Wellbeing EU Joint Action, 2017; Väilmaa et al., 2007), and evidence suggesting that loneliness in young people is related to PWB (Holt‐Lunstad et al., 2015; Matthews et al., 2019), the current study utilizes a large representative sample of older adolescents to explicitly test that relationship.

### 1.2 Moderators of loneliness and personal wellbeing

In addition to the need to understand the extent to which loneliness relates to PWB among young people, it is critical to uncover variation in that relationship. For effective intervention strategies or public health policies to be developed, it is necessary to understand the contextual factors that may either lessen or worsen the relationship between loneliness and PWB. For example, how might social support mitigate the negative effects of loneliness on well‐being? And, how might stronger neighborhood trust ease the suffering of those reporting loneliness?

To date, there is very little research examining factors that may moderate loneliness among young people. Research with school‐aged adolescents that explores loneliness and aspects of personal well‐being, such as mental health and life satisfaction, supports findings from work with older adults (Gow et al., 2007; Musich et al., 2015), showing how loneliness acts as a mediator between peer relationship difficulties and poor mental health (Corsano et al., 2006). Despite that work, there is limited evidence of contextual factors that might alleviate the negative effects of loneliness on the personal well‐being of young people.

A recent study (Moksnes et al., 2021) investigated gender as a moderator of loneliness, health, depression, anxiety, and mental wellbeing (assessed using the Warwick–Edinburgh Mental Well‐Being Scale, Tennant et al., 2007). The study found that, among young people aged 15–21 years, gender moderated the association between loneliness and depression and anxiety symptoms only. Our study advances previous research by investigating a number of factors that moderate the relationship between loneliness and PWB, to identify contexts that may ameliorate or exacerbate the negative relationship between loneliness and PWB among young people, thus providing novel insight into targets for intervention and public health policy development.

### 1.3 Theory

Framed by social‐ecological theory (Bronfenbrenner, 2005), PWB is the result of multiple, interacting domains of influence at the micro, meso, and macro‐level (e.g., demographic, social environment, and wider community factors). To adequately understand the drivers of PWB, social‐ecological theory posits that each of these domains must be simultaneously considered. Nested in this framework, the current study investigates correlates of PWB across a range of demographic, interpersonal, and community aspects of young people's lives. Additionally, given the interacting nature of these influences, we explicitly test which demographic, interpersonal, or community factors may alter, or moderate, the relationship between loneliness and PWB.

In doing so, this study illuminates how the relationship between loneliness and PWB differs dependent on various contexts (e.g., community relationships, and gender). Importantly, the study focuses on identifying demographic factors that may put a young person at particular risk of poor PWB, while examining modifiable factors at the interpersonal and community level that could be leveraged in intervention efforts (e.g., strategies to improve neighborly relations).

### 1.4 Study aims

The primary aims of the study were to (a) examine the extent to which loneliness is related to PWB in older young people specifically, and (b) determine how this relationship differs dependent on social‐ecological factors.

Specifically, this study aimed to answer the following research questions:

1. Is greater loneliness associated with poorer personal wellbeing among young people?
2. What are the key social‐ecological factors (i.e., demographic, interpersonal, and community factors) that predict personal wellbeing among young people?
3. Which social‐ecological factors moderate the association between loneliness and personal wellbeing among young people?

## 2 MATERIALS AND METHODS

### 2.1 Participants and data

We used data from the 2017–2018 wave of the Community Life Survey (CLS), resulting in a sample size of 965 young people aged 16–24. The CLS is an annual household survey conducted with around 5000 working‐aged adults (aged 16–64 years) resident in England. CLS covers a range of topics that are key to understanding society and local communities, including volunteering, civic engagement, and neighborhood characteristics, as well as health and wellbeing. Data were collected via self‐completion of the survey either online or if requested, using a paper version. PWB data has been routinely collected in the United Kingdom since 2011, but is repeated cross‐sectional in nature, rather than longitudinal (Allin & Hand 2017; Dolan & Metcalfe, 2012; Tinkler, 2015). Our outcome of interest, PWB, was previously termed “subjective wellbeing” in the data set and accompanying publications, but following public focus groups, was subsequently termed “personal wellbeing,” which was deemed to be simpler to understand (Office for National Statistics, 2017). All data are publicly available from the UK Data Service.

### 2.2 Measures

In this study, variables are categorized according to three social‐ecological domains: demographic, interpersonal, and community factors. A full list of variables in each domain is described in Table 1 and summarized below.

**Table 1:** Descriptive statistics

Domain	Variable info	Mean	SD	Scale info	Missingness (%)
Personal wellbeing	Overall life sat	6.871	2.088	0 (not at all satisfied) to 10 (completely satisfied)	0.73
	Happy yesterday	6.769	2.352	0 (not at all happy) to 10 (completely happy)	0.73
	Things you do in your life are worthwhile	6.916	2.201	0 (not at all worthwhile) to 10 (completely worthwhile)	1.05
Loneliness	Frequency feel lonely	2.853	1.159	1 (never) – 5 (often/always)	1.13
Socio demographic	Age category	1.575	0.495	1 = 16–19 years;	0
				2 = 20–24 years	
	Number of adults in household	3.142	0.977	1 (one) – 4 (4 or more)	0.14
	Sex	1.586	0.492	1 = male; 2 = female	0.10
				Male: *n* = 399, 41.35%	
				Female: *n* = 565, 58.55%	
	Cohabiting	1.859	0.349	1 (yes) – 2 (no)	21.97
	Care responsibilities	1.935	0.245	1 (yes) – 2 (no)	14.40
	Urban/Rural	1.087	0.282	1 (urban) – 2 (rural)	0
				Urban: *n* = 881, 91.25%	
				Rural: *n* = 84, 8.70%	
	Socioeconomic status—household income	2.445	1.786	1 (under £5,199)	23.21
				– 8 (£75,000 or more)	
	Working full time	1.505	0.500	1 = no	14.09
				2 = yes	
	Full time student	1.814	0.950	1 = yes	13.99
				0 = no	
	Self‐rated health	4.167	0.818	1 (very bad) – 5 (very good)	14.09
Interpersonal Factors (Contact frequency)	How often meet family and friends	5.471	1.808	1 (never) – 8 (more than once a day)	0.31
	How often speak on the phone or video with family members or friends	5.642	1.856	1 (never) – 8 (more than once a day)	0.31
	How often text or message friends	7.075	1.461	1 (never) – 8 (more than once a day)	0.52
Interpersonal Factors (Social support)	If I needed help, there are people who would be there for me	3.659	0.628	1 (definitely disagree)	0.10
				– 4 (definitely agree)	
	If I wanted company or to socialize, there are people I can call on	3.54	0.673	1 (definitely disagree)	0.31
				– 4 (definitely agree)	
	Is there anyone who you can really count on to listen to you when you need to talk	2.700	0.477	1, no, no one; 2 yes, one person; 3, yes, more than one person	14.20
Interpersonal Factors (Social network composition)	Proportion of friend same—age	1.981	0.837	1 (all the same) –	14.09
				4 (less than half)	
	Same religion	2.378	1.046	1 (all the same) –	16.17
				4 (less than half)	
	Same education	2.108	0.892	1 (all the same) –	14.20
				4 (less than half)	
	Same ethnicity	2.234	0.932	1 (all the same) –	14.04
				4 (less than half)	
Community	How often do you chat to your neighbors, more than to just say hello?	2.606	1.339	1 (never) – 5 (most days)	0.73
	Thinking about the people who live in this neighborhood, to what extent do you believe they can be trusted?	2.787	0.887	1 (none of the people) – 4 (many of the people)	1.14
	How strongly do you feel you belong to your immediate neighborhood?	2.508	0.874	1 (not at all strongly) – 4 (very strongly)	0.73

*Note*: Scale information and descriptive are based on final coding of items.

#### 2.2.1 Outcome

##### Personal wellbeing (PWB)

PWB was constructed as a composite measure based on 3 survey items: life satisfaction (“Overall, how satisfied are you with your life nowadays”); happiness yesterday (“Overall, how happy did you feel yesterday?”), and a sense that life is worthwhile (“Overall, to what extent do you feel the things you do in your life are worthwhile?”). Each item was scored from 1 to 10 by participants, with higher scores reflecting higher agreement with each statement, therefore, overall scores for personal wellbeing have a maximum of 30 (Cronbach's *α* = 0.88). The ONS measures PWB by collecting data on life satisfaction, sense of purpose in life, happiness, and anxiety (Office for National Statistics, 2021). Therefore, our measure of PWB closely maps onto this conceptualization.

An alternative composite measure of personal wellbeing was created which included self‐rated anxiety (included after reverse coding), however, this returned a lower α of 0.77, and therefore anxiety was removed to retain higher internal consistency.

#### 2.2.2 Demographic variables

The study controlled for a range of socio‐demographic factors including age category (16–19 years or 20–24 years), gender (*male or female*), whether participants were cohabiting (*yes/no*), number of adults in the household (1*–*4 *or more*), household income (*under £5,199*–*£75,000 or more*), resident in a rural or urban area (*rural/urban*), caring responsibilities (*yes/no*), whether studying full time (*yes/no*), working full time (*yes/no*), and overall rating of health (*very bad*—*very good*). Items retained original coding except for caring status, whether working full time, and self‐rated health. Carer and work status were recoded, respectively, so that a rating of 2 represented having caring responsibilities or being in full‐time work, and 1 represented no caring responsibilities or not being in work. Similarly, health was scored on a 5‐point scale, with higher scores representing better health.

#### 2.2.3 Interpersonal relationship variables

##### Loneliness

Loneliness was assessed based on a single item, a direct measure of loneliness (“How often do you feel lonely?”), to which participants responded from 1 (*often/always*) to 5 (*never*). This was reverse coded before analysis, so higher scores would represent higher levels of loneliness. The single‐item, direct measure of loneliness is widely used in epidemiological studies of loneliness (Newmyer et al., 2020; Shiovitz‐Ezra & Ayalon, 2012).

##### Social support

To assess for perceived social support, we selected three items, including measures of the extent to which there are people “who would be there for me,” and that if they wanted to socialize, there are “people I can call on,” with higher scores representing greater agreement. We also included an item measuring the extent to which participants believed that there are people “who you can really count on to listen,” which required recoding so that higher scores represented greater agreement.

##### Contact frequency

We investigated both social support and frequency of contact, with an awareness that these distinct social components may act differently on loneliness and PWB (Valtorta et al., 2016). To determine the impact of the frequency of contact with friends and family on PWB, we included three items. These included how often participants: *meet up in person with family or friends*; *speak on the phone or video call family and friends*; and, *exchange text messages with family or friends*. Each of these items was rated from 1 to 8 and required reverse coding so that higher scores represented the greater frequency of contact.

##### Social network composition

Participants indicated the proportion of their friends who were of the same race, age, education level, and faith. Higher scores represented a greater degree of heterogeneity, or diversity, among social networks.

#### 2.2.4 Community variables

##### Talk with neighbors

A variable that measured how often young people talked with their neighbors was included. It asked how often respondents “chat to neighbors, more than just to say hello.” The frequency of talking to neighbors was measured on a five‐point scale, with higher scores indicating more time spent interacting.

##### Trust people in the neighborhood

A variable measuring trust in the neighborhood was included, measured by asking respondents the extent to which “people living in this neighborhood can be trusted.” This was measured on a 4‐point scale, with higher scores indicating higher levels of trust.

##### Belong to neighborhood

A variable indicating how strongly respondents feel they “belong to their neighborhood” was also included, measured on a 4‐point scale, with higher scores indicating more belongingness.

### 2.3 Statistical analysis

Linear regression techniques were used to identify predictors of PWB across demographic, interpersonal, and community domains of influence, including the focal parameter of loneliness. Moderated linear regression was used to test which key factors altered the association between loneliness and PWB.

#### 2.3.1 Multiple linear regression

Multiple linear regression was used to assess the strength of the relationship between each predictor and PWB. A hierarchical approach was adopted to sequentially add social‐ecological domains of interest. Model one tested for a direct effect of loneliness only on PWB. Demographic factors, interpersonal factors, and community factors were added to subsequent models. Hierarchical regression was used rather than a stepwise approach, as this allowed for a theoretically driven process, in which subsequent variables were entered into the model based on key research considerations (e.g., adding additional social‐ecological domains of influence). Model assumptions for linear regression were tested, and no indications of multicollinearity (VIF < 0.10 and, bivariate correlations <0.80) were found. Additionally, Durbin Watson tests were conducted on all models with results close to two, indicating that assumptions of linearity and homoscedasticity were met.

#### 2.3.2 Moderated multiple regression

Moderated multiple regression was employed to examine the effect of specified moderating variables on the relationship between PWB and loneliness among young people. A number of individual moderation models were tested through the use of interaction effects while controlling for other predictors in the model. Moderators tested included key demographic factors (i.e., age, gender, health, Socioeconomic status (SES), rural vs. urban), as well as all items used to assess interpersonal relationship factors and community factors. A significant interaction term indicated a significant moderating effect was present.

### 2.4 Missing data

Levels of missing data are listed in Table 1. Missing data were handled via multiple imputation chained equations (Raghunathan et al., 2001) using the MICE package (van Buuren & Groothuis‐Oudshoorn, 2011) for RStudio. We performed 20 imputations of the data set. Data were imputed via the classification and regression trees (CART) method (Breiman et al., 1984). This method of imputation is robust against outliers and multicollinearity and is suitable for fitting interactions in regression models (Burgette & Reiter, 2010). All analyses were conducted in RStudio Version 1.3.1093.

## 3 RESULTS

### 3.1 Descriptive results

Table 1 displays descriptive statistics for the sample. Approximately 58% of the sample were female. On average, scores for loneliness were mid‐range (mean = 2.85, range: 1–5), with the mean score falling between ratings of “hardly ever” and “occasionally” feeling lonely. Those indicating that they felt lonely “often or always” (*n* = 80, 8.29%), or “sometimes” (*n* = 250, 22.28%) constituted 30.57% of the sample population. This is in line with prevalence rates of loneliness among young people reported elsewhere, for example, in a study sampling 2066 18‐year‐olds, 5%–7% reported feeling lonely often, and 23%–31% some of the time (Matthews et al., 2019). Around 91% of the sample reside in urban areas.

### 3.2 Multiple linear regression

Results from multiple linear regression can be seen in Table 2. An initial model using loneliness as a single predictor of PWB identified a negative association between loneliness and PWB (*b* = −2.374, SE = 0.151). This relationship remained for all subsequent models. Model 2 included demographic factors, in which being a carer (*b* = −1.193, SE = 0.723), a full‐time student (*b* = 1.367, SE = 0.481), and ratings of overall health (*b* = 2.395, SE = 0.236) were associated with PWB. These effects persisted in all subsequent models. Interpersonal relationship factors were added in model 3, and of those only a sense of being able to count on friends was significantly associated with PWB (*b* = 1.221, SE = 0.397). Results from the final model (Model 4), which controlled for loneliness, demographic factors, interpersonal factors, and community factors, showed a range of characteristics were associated with PWB. Loneliness demonstrated a significant negative effect on PWB (*b* = −1.679, SE = 0.166). Having caring responsibilities for a family member with a long‐standing illness was also negatively associated with PWB (*b* = −1.942, SE = 0.719). Demographic factors positively associated with PWB included being a full‐time student (*b* = 1.125, SE = 0.479), and having greater self‐rated health (*b* = 2.022, SE = 0.239).

**Table 2:** Multiple linear regression output (dependent variable–personal wellbeing)

Domain	Subdomain		(1)	(2)	(3)	(4)
		**Loneliness**	−**2.374** *** **(0.151)**	−**1.898** *** **(0.164)**	−**1.707** *** **(0.168)**	−**1.679** *** **(0.166)**
Demographic factors		Age		0.335 (0.407)	0.336 (0.406)	0.638 (0.402)
		No. adults in house		−0.120 (0.256)	−0.101 (0.253)	−0.126 (0.248)
		Sex		0.615 (0.367)	0.458 (0.376)	0.416 (0.369)
		Cohabiting		−0.621 (0.570)	−0.575 (0.597)	−0.692 (0.586)
		**Carer**		−**1.913 (0.723)** **	−**1.738** * **(0.724)**	−**1.942** ** **(0.719)**
		Rural		−0.060 (0.597)	−0.047 (0.598)	−0.103 (0.601)
		Socioeconomic status		0.018 (0.146)	−0.013 (0.143)	−0.144 (0.140)
		Working		0.540 (0.415)	0.259 (0.421)	0.135 (0.414)
		**Student**		**1.367** ** **(0.481)**	**1.216** * **(0.485)**	**1.125** * **(0.479)**
		**Overall health**		**2.395** *** **(0.236)**	**2.197** *** **(0.239)**	**2.022** *** **(0.239)**
Interpersonal factors	Contact frequency	How often meet friends			0.048 (0.117)	0.001 (0.115)
		Speak on phone			0.011 (0.112)	−0.002 (0.112)
		Text friends			−0.033 (0.138)	0.014 (0.136)
	Social support	People there for me			0.418 (0.394)	0.419 (0.388)
		Can call on friends			0.401 (0.394)	0.283 (0.386)
		**Count on friends**			**1.221** ** **(0.397)**	**1.156** ** **(0.390)**
	Social network composition	Same age			0.437 (0.267)	0.415 (0.262)
		Same faith			−0.181 (0.199)	−0.081 (0.195)
		Same race			0.267 (0.220)	0.324 (0.216)
		Same education level			−0.125 (0.243)	−0.169 (0.239)
Community factors		**Chat to neighbors**				**0.327** * **(0.153)**
		**Trust in neighborhood**				**0.652** ** **(0.221)**
		**Belong to neighborhood**				**0.500** * **(0.225)**
		Intercept	**27.281** *** **(0.468)**	**16.256** *** **(2.340)**	**9.870** *** **(2.735)**	**7.340** ** **(2.723)**
		*R* ^2^	0.209	0.334	0.361	0.389
		Adjusted *R* ^2^	0.208	0.324	0.343	0.368

* *p* < 0.05.

** *p* < 0.01.

*** *p* < 0.001.

In relation to interpersonal relationship factors, only being able to count on friends and family to listen if they needed to talk (*b* = 1.156, SE = 0.390) showed a (positive) association with PWB. As a supplemental check for the interpersonal relationship factors, we tested a model in which all three social support items were modeled as a composite variable (Cronbach's α = 0.79). This was strongly associated with PWB. The final model therefore contains individual social support items, to identify specific dimensions of social support which are associated with adolescent wellbeing. All other interpersonal factors demonstrated no significant association with wellbeing, therefore similar supplemental checks were not conducted for these items. Community factors were consistently, positively associated with PWB. That is, young people who reported higher levels of chatting to neighbors (*b* = 0.327, SE = 0.153), higher belief that people in the neighborhood could be trusted (*b* = 0.652, SE = 0.221), or a greater sense of belonging to their neighborhood (*b* = 0.500, SE = 0.225) had higher levels of PWB.

### 3.3 Moderated linear regression

**Figure 1:**
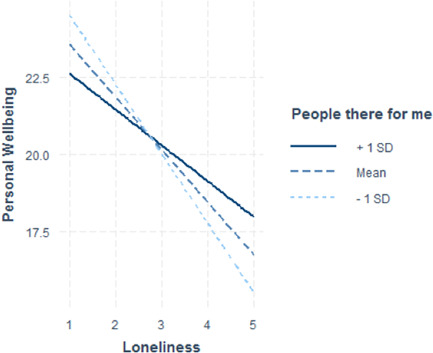
Moderating effect of interpersonal factor: “If I needed help, there are people who would be there for me”

**Figure 2:**
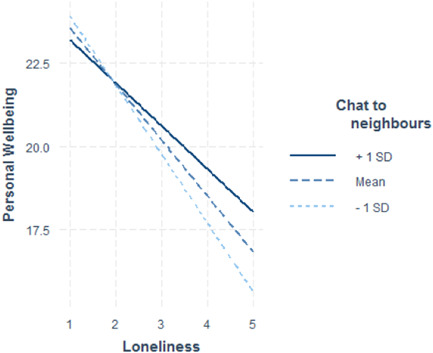
Moderating effect of community factor: “How often do you chat to your neighbors, more than to just say hello?”

**Figure 3:**
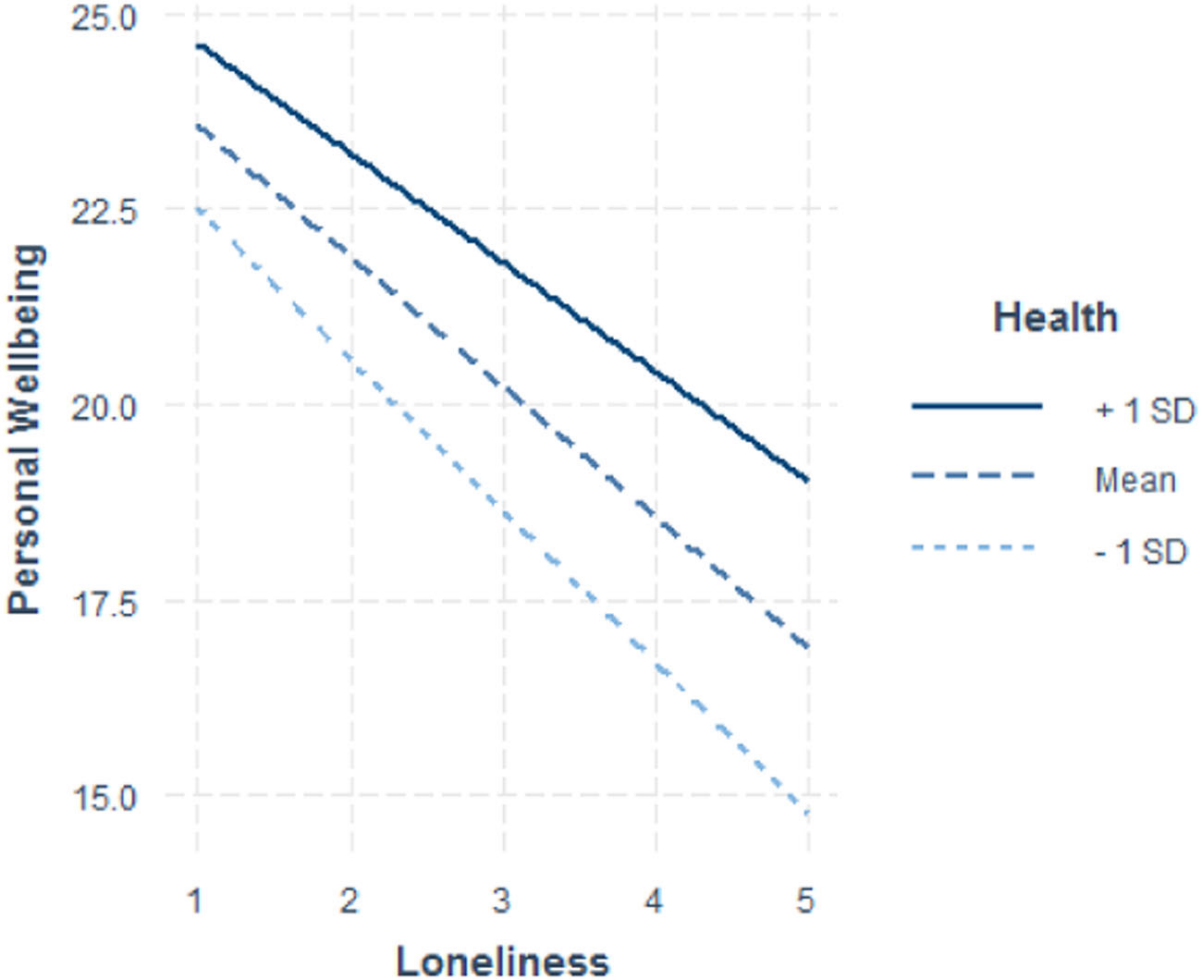
Nonsignificant moderating effect of health on loneliness and personal wellbeing

Of all moderators tested (demographic factors: age, SES, gender, urban vs. rural, health; all contact frequency, social support, social network composition, and community items), only two showed a significant moderating effect on the relationship between loneliness and personal wellbeing (see Table 3; Figures 1, 2, 3). These included (1) the extent to which young people believe that if they needed help, there are people who would be there for them (*b* = 0.867, SE = 0.341), and (2) how often young people chat with their neighbors (*b* = 0.302, SE = 0.130). Both moderators demonstrated a positive interaction, meaning that for young people who reported the most loneliness, those who also reported either a greater sense that people are there for them or more frequent talking with neighbors, had higher PWB than equally lonely peers. Therefore, these are protective factors against the negative effects of loneliness on PWB. Self‐rated health was marginally significant (*b* = 0.331, SE = 0.169, *p* = 0.051), but also demonstrated a protective effect against the negative effects of loneliness on PWB. As can be seen in Figure 3, those with higher rated health had higher levels of PWB at all levels of loneliness.

**Table 3:** Moderated multiple linear regression output: moderators of personal well‐being and loneliness

Domain	Subdomain	Moderator	People there for me	Chat to neighbors
		Loneliness	−**2.947** *** **(0.878)**	−**1.868** ** **(0.571)**
Demographic factors		Age	0.629 (0.401)	0.628 (0.401)
		No. adults in house	−0.108 (0.249)	−0.144 (0.248)
		Sex	0.373 (0.368)	0.412 (0.369)
		Cohabiting	−0.700 (0.584)	−0.707 (0.587)
		Carer	−**1.951** ** **(0.601)**	−**1.953** ** **(0.718)**
		Rural	−0.182 (0.601)	−0.222 (0.602)
		Socioeconomic status	−0.013 (0.140)	−0.028 (0.140)
		Working	0.114 (0.413)	0.087 (0.413)
		Student	**1.128** * **(0.475)**	**1.090** * **(0.476)**
		Overall health	**2.017** *** **(0.238)**	**2.026** *** **(0.238)**
Interpersonal factors	Contact frequency	How often meet friends	−0.002 (0.115)	0.0119 (0.115)
		Speak on phone	0.015 (0.111)	−0.014 (0.112)
		Text friends	0.0154 (0.136)	0.044 (0.137)
	Social support	People there for me	−**2.290** * **(1.151)**	0.478 (0.389)
		Can call on friends	0.846 (1.086)	**0.189 (0.388)**
		Count on friends	**2.554** * **(1.067)**	**1.167** ** **(0.390)**
	Social network composition	Same age	0.439 (0.261)	0.420 (0.262)
		Same faith	−0.096 (0.195)	−0.113 (0.195)
		Same race	0.318 (0.216)	0.348 (0.216)
		Same education level	−0.153 (0.239)	−0.160 (0.240)
Community factors		Chat to neighbors	0.335 (0.1527)	‐0.554 (0.408)
		Trust in neighborhood	**0.621** ** **(0.221)**	0.832 (0.547)
		Belong to neighborhood	**0.509** * **(0.225)**	1.014 (0.576)
		Interaction	**0.867** * **(0.341)**	**0.302** * **(0.130)**
		Intercept	**11.626** ** **(3.962)**	**8.068** * **(3.167)**
		*R* ^2^	0.395	0.393
		Adjusted *R* ^2^	0.373	0.371

* *p* < 0.05.

** *p* < 0.01.

*** *p* < 0.001.

## 4 DISCUSSION

This study aimed to establish the association between loneliness and PWB among young people aged 16–24 years and identify social‐ecological moderators of that relationship. Importantly, we examined demographic factors that may heighten the risk of poor PWB, and modifiable interpersonal and community factors. The study advances the literature by identifying contextual factors that alleviate the negative relationship between loneliness and the personal well‐being of young people, thus providing important targets for public health strategies targeting youth loneliness and wellbeing.

### 4.1 Predictors of personal wellbeing

Across all models, loneliness was consistently associated with a decrease in PWB. In practical terms, each one‐point increase on the loneliness scale (e.g., from “hardly ever” lonely to “occasionally” lonely), was associated with a decrease of 1.68 on the PWB scale. This is unsurprising given the literature that notes significant, negative impacts of youth loneliness on related factors such as poor mental health (Matthews et al., 2019) and physical health (Eccles et al., 2020).

Additionally, at the interpersonal level, having friends you can count on was predictive of PWB among young people. This makes it particularly vital to understand any perceived lack of supportive relationships among young people, who are both more likely to experience loneliness and, as our study shows, likely to show improved wellbeing in the presence of supportive social relationships.

We also identified that community factors, such as frequently talking to neighbors, a greater degree of trust in those living in the neighborhood, and an increased sense of belonging to the neighborhood, were important in determining increased PWB. In contrast to this, we found no association between how frequently participants contacted friends and family and PWB, suggesting that quality of social support may be more important for wellbeing than the frequency of social contact. These findings align with previous research that found that having poor quality social relationships was associated with poor psychological health, while a low quantity of social connections was not (Hyland et al., 2019).

Furthermore, we found that the composition of young people's social networks (i.e., the extent to which their friends were of the same age, race, education, and faith) was not an important factor in predicting wellbeing. Again, it seems it is not necessarily the objective characteristics of one's social network that matters, but rather, the perceived quality of these relationships that is important in terms of improved PWB. This is supportive of previous research which identifies that social network size is less important for well‐being than the emotional quality of close relationships in adults across the lifespan (Bruine de Bruin et al., 2020).

### 4.2 Moderators of loneliness and personal wellbeing

Our study identifies predictors of wellbeing among a contemporary sample of young people, but also, it identifies potential protective factors against the deleterious effects of loneliness on PWB. By testing for moderators of loneliness and PWB, we were able to establish that supportive relationship, and involvement with others in the community were important protective factors for reducing the negative consequences of loneliness on young people's PWB. Our results demonstrated that amongst young people reporting the most loneliness, those with a greater perception that people were ‘there for’ them had higher PWB than equally lonely peers with a reduced sense of this emotional support. Similarly, for the loneliest young people, those who reported increased communication with neighbors had higher PWB than equally lonely peers. These findings suggest that social‐emotional connectedness is a key factor in protecting the loneliest young people's well‐being.

Our results suggest that it is important to develop interventions that promote young people's involvement in the community, or that foster and promote meaningful social interactions. Social prescribing has been demonstrated to reduce loneliness among adult populations (Foster et al., 2020; Kellezi et al., 2019), and improve wellbeing (Bickerdike et al., 2017; Grant et al., 2000; Moffatt et al., 2017) and may be an effective method of increasing engagement in the community for young people. Social prescribing is part of the UK's 2018 Loneliness Strategy, and while demonstrated to be effective in older adults, less is known of its efficacy in younger populations. To reach younger populations, it may be important to consider educational‐based approaches in schools and further education settings (Siva, 2020); ideally, those would be peer‐led and based on participatory principles to maximize efficacy and engagement. Our results suggest that social prescribing at a local level, or other interventions that foster closer connections with those in the immediate neighborhood, may prove fruitful in reducing the negative effects of loneliness among those aged 16–24 years.

### 4.3 Limitations and future research

It is important to note some limitations of our study. First, due to data availability, we used a single‐item measure of loneliness. This is known as a direct measure of loneliness because it specifically makes use of the word “lonely.” This represents a phenomenological approach that allows the respondent to determine what the concept of loneliness means to them personally (Nicolaisen & Thorsen, 2014). While commonly used in epidemiological and survey research, it has been noted that due to the stigma associated with experiencing loneliness, direct measures can result in under‐reporting of loneliness, particularly so among male respondents (de Jong Gierveld et al., 2006). However, research establishing loneliness as a stigmatized condition may now be outdated (Kerr & Stanley, 2021) and contemporary societal attitudes toward loneliness are not necessarily negative or stigmatizing (Barreto et al., 2022). As the data used in the current study were collected in 2017–2018, it is likely in the context of less societal stigma toward loneliness, and therefore the risk of under‐reporting with a direct measure may not be as likely. We also acknowledge that a limitation of the direct, single measure of loneliness is that it may fail to capture the multidimensional nature of loneliness (Goossens et al., 2009), and therefore may not capture every facet of loneliness in young adulthood.

Second, due to the availability of data, we modeled age as a categorical variable (i.e., 16–19 and 20–24 years). This allowed us to broadly examine whether adolescence (16–19 years) or young adulthood (20–24 years) was associated with increased PWB and whether these developmental categories moderated the relationship between loneliness and PWB. However, we were not able to take a more nuanced approach in examining the effect of age on PWB and its association with loneliness. Although research exists that examines loneliness across the life course (e.g., Qualter et al., 2015), there is comparatively less which looks at changes specifically during later adolescence and young adulthood (i.e., 16–24 years) and how this may consequently impact PWB. This merits future investigation to gain a more detailed understanding of the interaction between loneliness and wellbeing among young people, particularly during key transition points such as leaving school or home, thus allowing for the development of targeted, timely intervention strategies.

Third, our selection of moderator variables was constrained by data contained within the secondary data set. While we were able to examine the moderating effect of some individual, interpersonal, and community factors, it may be that there are additional interpersonal or community‐level characteristics that protect against the negative effects of loneliness on the PWB of young people. In relation to individual‐level factors, data available in the current study lacked information on participants' sexual orientation. It is known that loneliness may be higher among young people of minority sexual orientation (Eres et al., 2020; Marquez et al., 2022). Compared to heterosexual or cisgender peers, LGBTQI + youth are more likely to experience factors which may increase loneliness and reduce wellbeings, such as family rejection, bullying, and violence (Gamarel et al., 2014) and more widespread cultural stigma (Barreto et al., 2022). It is therefore important to pay close attention to the association between loneliness and well‐being among transgender and LGBTQI + young people.

Additionally, our analysis was conducted in a predominately non‐rural population. It is known that living in remote and rural areas is a significant risk factor for increased social isolation and loneliness among older adults (Hart, 2016; Henning‐Smith et al., 2018). However, more research is needed to identify the specific risk factors for reduced PWB among rural young people, as well as moderating factors that may protect against the negative effects of loneliness on PWB.

Finally, our data reflects responses collected from young people living in England only. It may be that associations between loneliness and PWB, and any moderating effects on these, may differ in other socio‐cultural or geographical contexts.

## 5 CONCLUSIONS

This study is novel in providing (1) insight into key social‐ecological factors associated with PWB among young people, and (2) identifying which of these factors may alter associations between loneliness and PWB. Our findings are likely to be especially helpful in the development of targeted public health interventions seeking to mitigate the negative effects of youth loneliness on personal wellbeing, as results indicate that community and interpersonal relationship factors may have an important role in enhancing wellbeing among lonely young people. Additionally, results suggest that individual characteristics, such as having caring responsibilities within the home, or poorer physical health, serve as risk factors for PWB. Thus, our study highlights the need for these vulnerabilities to be acknowledged during the development of interventions aiming to improve the well‐being of young people.

## CONFLICTS OF INTEREST

The authors declare no conflicts of interest.

## ETHICS STATEMENT

This study used publicly available secondary data collected by Kantar Public, on behalf of the government Department for Digital, Culture, Media, and Sport which retain responsibility for ethical approval of the survey.

## Data Availability

The data underlying this article are publicly available in the UK Data Service, Community Life Survey 2017–18, at 10.5255/UKDA‐SN‐8478‐1.
